# Is there any difference between Eastern and Western clinical practice guidelines in management of gastric cancer?

**DOI:** 10.1002/cnr2.2076

**Published:** 2024-05-06

**Authors:** Amirmohsen Jalaeefar, Seyedeh Zahra Mousavi, Mohammad Shirkhoda, Habibollah Mahmoodzadeh, Narjes Mohammadzadeh, Amirsina Sharifi

**Affiliations:** ^1^ Department of Surgery, Subdivision of Surgical Oncology, Cancer Institute Tehran University of Medical Sciences Tehran Iran; ^2^ Cancer Research Center of Cancer Institute, Tehran University of Medical Sciences Tehran Iran; ^3^ Department of Surgery, Imam Khomeini Hospital Complex Tehran University of Medical Sciences Tehran Iran; ^4^ Sina Trauma and Surgery Research Center Tehran University of Medical Sciences Tehran Iran

**Keywords:** cancer management, clinical practice guideline, gastric cancer

## Abstract

**Background and Recent findings:**

Gastric cancer (GC) has been known as one of the most common causes of cancer mortality both in Western and Eastern countries. However, there might be differences between how it is managed in different countries. Thus, we aimed to investigate these differences.

**Materials and Methods:**

The most well‐known clinical guidelines in field of GC management including Korean GC Association (KGCA), Japanese GC Association (JGCA), National Comprehensive Cancer Network (NCCN), European Society for Medical Oncology (ESMO), British Society of Gastroenterology (BSG), and National Institute for health and Care Excellence (NICE) have been reviewed.

**Results:**

The contents of these guidelines were categorized under eight headings including (1) genetic predisposition, (2) prevention, (3) management of gastric polyp, atrophy, dysplasia and metaplasia, (4) diagnosis, (5) pathology and molecular biology, (6) treatment, (7) supportive and palliative care, and (8) follow up. Difference in each section was discussed.

**Conclusion:**

Considering KGCA and JGCA as Eastern and NCCN, ESMO, BSG, and NICE as Western guidelines, it is revealed that both sets of guidelines share common practices such as prioritizing comprehensive diagnostic evaluations, personalizing treatment plans, and palliative care. However, main differences can be seen in treatment regimens, the adoption of newer therapies like immunotherapy, and the utilization of emerging techniques such as HIPEC. These differences reflect the diverse clinical landscapes, research focuses, and healthcare systems within these regions.

## 1 INTRODUCTION

Gastric cancer (GC) is the sixth most common malignancy with more than one million new cases worldwide and the third cause of cancer death.^1^, ^2^ GC is classified based on tumor location, distinguishing between proximal GC (including the cardia and esophagogastric junction [GEJ]) and distal GC (located in the antrum/pylorus). These subtypes have distinct etiologies, with proximal GC linked to esophageal reflux and obesity, prevalent in Western countries. Epstein–Barr virus (EBV) positive GC is more common in the upper stomach and body, exhibiting similar prevalence in Asia to elsewhere.^3^, ^4^ Distal GC is primarily associated with chronic *Helicobacter pylori* infection. Globally, distal gastric adenocarcinoma has double the incidence of proximal gastric adenocarcinoma, though this ratio varies globally and regionally. Notably, Japan has reported an increased incidence of proximal esophagogastric junction (EGJ) cancers.^3^, ^5^, ^6^ The highest rates for both subtypes are observed in Eastern/Southeastern Asia, with factors such as *H. pylori* infection, dietary habits, lifestyle, and genetics considered contributory to GC development.^7^, ^8^

As it was mentioned, neither the prevalence nor the attributed deaths are the same globally. The regions with the highest rates are Eastern Asia, Central and Eastern Europe, and South America.^9^ On the other hand, Western Europe and North America have witnessed a decline in GC prevalence and death over the past 60 years, and by contrast a relative increase in esophageal adenocarcinoma was observed.^10^ An enormous amount of data is generated each year in GC. This large volume of studies will finally end up in guidelines after being filtered for methodical quality. These guidelines aim to accelerate decision‐making while adhering to the most current evidence‐based practices.

Though it is anticipated that clinicians take these guidelines into consideration, it is equally important to respect the individual needs, preferences, and values of their patients. Also, the availability and the cost of what clinicians suggest should be accustomed to the national and local resources. So, there would be a gap between gold standard and practical treatment available in cancer centers, especially in developing countries. The objective of this study was to contrast the most recently published guidelines in management of GC to find if there is any difference in diagnosing, staging, and treatment of GC and provide a spectrum of choices to approach patients with GC.

## 2 MATERIALS AND METHODS

The choice of guidelines was predominantly guided by their significance and influence on the body of English literature concerning GC. Therefore, guidelines published by the most sophisticated organizations were retrieved. Moreover, to ensure comparability in content and time frame, only guidelines released after January 1, 2018, incorporating tumor classification based on the eighth edition of the American Joint Committee TNM system (2016)^11^ were considered for inclusion. Using this method, we gathered six different guidelines (Table 1).

**TABLE 1:** Gastric cancer (GC) guidelines used in this study.

Country	Organization	Title	Publication year
Korea	Korean GC Association (KGCA)	Korean Practice Guidelines for GC 2022: An Evidence‐based, Multidisciplinary Approach^12^	2023
Japan	Japanese GC Association (JGCA)	Japanese GC Treatment Guidelines 2021 (6th ed)^13^	2022
USA	National Comprehensive Cancer Network (NCCN)	GC^14^	2022
Europe	European Society for Medical Oncology (ESMO)	GC: ESMO Clinical Practice Guideline for diagnosis, treatment and follow‐up^15^	2022
UK	British Society of Gastroenterology (BSG)	British Society of Gastroenterology guidelines on the diagnosis and management of patients at risk of gastric adenocarcinoma^16^	2019
UK	National Institute for health and Care Excellence (NICE)	Oesophago‐GC: assessment and management in adults^17^	2018

In each section we defined key questions attributed to the subject and tried to answer relevant questions based on specific content of each guideline. Table 1 shows each guideline characteristics.

## 3 RESULTS

### 3.1 Genetic predisposition

#### 3.1.1 Who should consider consulting a specialist in cancer genetic?

Based on the information provided by the National Comprehensive Cancer Network (NCCN) and British Society of Gastroenterology (BSG) guidelines, individuals who should be referred to a cancer genetics professional for evaluation of familial or hereditary GC are as follows:

- An individual diagnosed with GC before age 40.
- An individual diagnosed with GC before age 50 who had one first‐ or second‐degree relative affected with GC.
- An individual diagnosed with GC at any age who has 2 or more first‐ or second‐degree relatives affected with GC.
- An individual diagnosed with GC and breast cancer with one diagnosis before age 50.
- An individual diagnosed with GC at any age and a family history of breast cancer in a first‐ or second‐degree relative diagnosed before age 50.
- An individual diagnosed with GC at any age and a family history of juvenile polyps or gastrointestinal polyposis.
- An individual diagnosed with GC at any age and a family history of cancers linked to Lynch syndrome (colorectal, endometrial, small bowel, or urinary tract cancer).

### 3.2 Prevention

#### 3.2.1 Who should be screened for GC?

Each guideline puts forth distinct criteria for screening individuals for GC. According to Korean GC Association (KGCA), it is advised to screen all individuals aged 40 years or older every 2 years, whereas European Society for Medical Oncology (ESMO) and BSG focus on specific high‐risk groups, including individuals with conditions like intestinal metaplasia, family history of GC, or other risk factors, and recommend endoscopic surveillance every 3 years for these patients. The guidelines also stress the significance of taking into account risk factors, including age, gender, cigarette smoking history, pernicious anemia, and family history of GC, when deciding on screening and surveillance protocols.

## 4 MANAGEMENT OF GASTRIC POLYP, ATROPHY, DYSPLASIA, AND METAPLASIA

### 4.1 How gastric polyps should be managed?

According to BSG recommendations, it is crucial to meticulously record the number of gastric polyps, their location, and the size of the largest one. Any gastric polyps that are not fundic gland polyps (FGPs) should undergo biopsy for histopathological evaluation. If adenomas or hyperplastic polyps are identified, the surrounding mucosa should be examined endoscopically for indicators of gastric atrophy, gastric intestinal metaplasia, *H. pylori* presence, and synchronous neoplasia. Resection of all adenomas is advised when it is deemed clinically appropriate and safe. Subsequent gastroscopy should be conducted at the 1‐year mark after complete endoscopic excision of adenomas, followed by annual surveillance gastroscopy as deemed necessary. Hyperplastic polyps exceeding 1 cm in size, those with a pedunculated morphology, or those causing symptoms (such as obstruction or bleeding) should be excised. If *H. pylori* is present, it should be eradicated before considering re‐evaluation for endoscopic therapy. In cases of diagnostic uncertainty following white light examination, enhanced endoscopic imaging may be utilized to aid in the characterization of gastric polyps.

## 5 DIAGNOSIS

### 5.1 What role does fluorodeoxyglucose positron emission tomography/computed tomography play in the staging of GC?

The role of fluorodeoxyglucose positron emission tomography/computed tomography (FDG PET/CT) in the staging of GC can be summarized as follows:

- KGCA: FDG PET/CT serves as a supplementary diagnostic tool, used in conjunction with diagnostic CT, for the staging workup of GC. It exhibits high specificity but lower sensitivity in the detection of lymph node metastasis and distant metastasis. Additionally, FDG PET/CT proves valuable in distinguishing cancer recurrence from inflammatory and postoperative changes.
- Japanese GC Association (JGCA): The recommendation for not performing a PET‐CT scan for GC staging is considered weak.
- NCCN: FDG‐PET/CT displays a lower level of accuracy compared with CT scans, particularly in cases of diffuse and mucinous tumor types. While it demonstrates enhanced specificity in detecting local lymph node involvement, its sensitivity is comparatively lower. When used alongside CT, FDG‐PET/CT can enhance preoperative staging accuracy. However, it does not substitute for staging laparoscopy in peritoneal disease detection.
- ESMO: FDG PET/CT may enhance staging by identifying affected lymph nodes or metastatic disease. Nonetheless, it may not yield informative results in patients with mucinous or diffuse tumors due to reduced tracer uptake. As a result, FDG PET/CT is not routinely recommended for GC staging.
- NCIE: F‐18 FDG PET‐CT is recommended for individuals with esophageal and gastro‐esophageal junctional tumors suitable for radical treatment (excluding T1a tumors). In cases of GC, F‐18 FDG PET‐CT should only be contemplated when metastatic disease is suspected, aiding in guiding ongoing management.

### 5.2 What is the role of staging laparoscopy and peritoneal washings in GC?

The role of staging laparoscopy and peritoneal washings in GC can be summarized as follows based on each guide line:

- JGCA: For patients with advanced GC likely to have peritoneal metastasis, staging laparoscopy is weakly recommended for treatment planning.
- NCCN: Laparoscopy can be a valuable tool for detecting radiographically occult metastatic disease, especially in cases of locally advanced (cT3) tumors or lymph node involvement (cN+) seen on preoperative imaging, in patients being considered for surgical resection without preoperative therapy.
- ESMO: All potentially resectable patients with stage IB‐III GC should undergo laparoscopy and peritoneal washing. This is especially beneficial for patients with T3/T4 disease and poorly cohesive tumors. Documenting peritoneal metastases according to the Peritoneal Carcinomatosis Index (PCI) is recommended.
- National Institute for health and Care Excellence (NICE): Staging laparoscopy should be considered to guide ongoing management, specifically for individuals with esophageal or gastro‐esophageal junctional cancer. It is advised to offer staging laparoscopy to all individuals with potentially curable GC.

## 6 PATHOLOGY AND MOLECULAR BIOLOGY

### 6.1 What is the recommended GC histopathological classification scheme?

According to the KGCA guideline, the preferred histopathological classification system for GC is based on the World Health Organization's (WHO) fifth edition classification of digestive tumors. Additionally, the Lauren classification can be employed for resected specimens, including those obtained through endoscopic submucosal dissection (ESD). However, the NCCN recommends using the College of American Pathologists Cancer Protocols for reporting pathological findings. The differentiation of gastric adenocarcinomas into intestinal or diffuse types may hold significance for treatment decisions, particularly as intestinal type cancers may be more prone to overexpressing HER2.

## 7 TREATMENT

### 7.1 Endoscopic treatment

#### 7.1.1 What is the eligibility criteria for endoscopic mucosal resection and ESD in GC?

It is important to note that endoscopic mucosal resection (EMR) and ESD are appropriate for early‐stage GCs (T1a) that are limited to the mucosa or have superficial submucosal invasion. Each guideline has its own indication for EMR and ESD which are as follow:

- KGCA
  ○ EMR is advised for well or moderately differentiated tubular or papillary early GCs that meet specific criteria, including tumor size of 2 cm or smaller, presence of mucosal cancer, and absence of ulceration.
  ○ ESD is indicated for larger well or moderately differentiated tubular or papillary early GCs with specific criteria like tumor size >2 cm (without ulcer) or tumor size ≤3 cm (with ulcer).
- JGCA
  ○ Absolute indications for EMR or ESD include: Differentiated‐type adenocarcinoma without ulcer, depth of invasion clinically T1a, and diameter ≤2 cm.
  ○ Additional ESD indications: Differentiated‐type adenocarcinoma with ulcer (diameter ≤3 cm) and undifferentiated‐type adenocarcinoma (diameter ≤2 cm).
- NCCN
  ○ EMR or ESD adequate when: Lesion ≤2 cm, well or moderately well differentiated, no penetration beyond superficial submucosa, no lymphovascular invasion, and clear margins.
  ○ ESD more effective than EMR for small early‐stage GC, but higher risk of complications.
- ESMO
  ○ EMR recommended for very early GCs (T1a) meeting specific criteria: Confined to mucosa, well‐differentiated G1‐2, ≤2 cm, and non‐ulcerated.
  ○ Expanded criteria consider size, depth of invasion, and grade of differentiation for potential endoscopic resection.
- BSG
  ○ EMR for lesions ≤10 mm, ESD for lesions >10 mm (en bloc excision).
  ○ Complete (R0) endoscopic resection of gastric dysplasia and early gastric adenocarcinoma with the following features should be considered as curative: low‐grade dysplasia (LGD), high‐grade dysplasia (HGD), well or moderately differentiated intramucosal adenocarcinoma irrespective of size and without ulceration, well or moderately differentiated submucosal adenocarcinoma, less than 3.0 cm in size if ulcerated, well or moderately differentiated submucosal adenocarcinoma, less than 3.0 cm in size, with superficial submucosal invasion, and poorly differentiated intramucosal adenocarcinoma with a maximum size of 2.0 cm.

### 7.2 Surgery (gastrectomy)

#### 7.2.1 What is the preferred treatment for stage Ia GC?

The preferred treatment for stage Ia GC (early‐stage, limited to the mucosa or submucosa) depends on the specific characteristics of the tumor and its location. Different guidelines provide different recommendations:

- JGCA
  ○ For cT1N0 tumors (stage Ia), the choice of gastric resection can be influenced by the tumor's location: (a) Pylorus‐preserving gastrectomy (PPG): this is suitable for tumors in the middle portion of the stomach, where the distal tumor border is at least 4 cm proximal from the pylorus. (b) Proximal gastrectomy (PG): this is recommended for proximal tumors where more than half of the distal stomach can be preserved. (c) Local resection of the stomach and segmental gastrectomy should still be considered as experimental treatments.
- NCCN
  ○ Patients with Tis (carcinoma in situ) or T1 limited to the mucosa (T1a) may be eligible for EMR or ESD provided they meet the necessary criteria and receive treatment at specialized, experienced centers.
- ESMO
  ○ T1 tumors not meeting the criteria for endoscopic resection necessitate surgery, albeit a less extensive procedure compared with other stages of GC.
  ○ Lymph node dissection for T1 tumors may be limited to perigastric lymph nodes and may encompass local N2 nodes (D1+ lymphadenectomy). The specific nodal groups dissected may vary based on the site of the cancer.

#### 7.2.2 What is the preferred treatment for stage IB‐III GC?

The preferred treatment for stage IB‐III GC (locally advanced disease) typically involves radical gastrectomy with lymphadenectomy. The specific surgical approach may vary depending on the tumor stage and location, and recommendations from different guidelines may slightly differ:

- JGCA
  ○ In cases of clinically node‐positive (cN+) or T2–T4a tumors, the standard surgical options are either total gastrectomy or distal gastrectomy.
  ○ The choice between distal gastrectomy and total gastrectomy depends on whether a satisfactory proximal resection margin can be achieved. Distal gastrectomy is preferred in such cases. Total gastrectomy, on the other hand, is the preferred option when obtaining a clean proximal resection margin is not feasible, or when the tumor involves the pancreas (necessitating pancreaticosplenectomy) or is located along the greater curvature (necessitating splenectomy).
  ○ In cases of adenocarcinoma of the GEJ, PG is also a viable option to consider.
- NCCN
  ○ For tumors with a T stage ranging from T1b to T3, it is recommended to perform a thorough gastric resection to ensure negative microscopic margins, coupled with lymphadenectomy.
  ○ In cases of T4b tumors with invasion into adjacent structures, it is imperative to conduct an en bloc resection of the involved structures.
- ESMO
  ○ Radical gastrectomy is the recommended treatment for stage IB‐III disease.
  ○ There has been significant debate regarding the extent of nodal dissection during radical gastrectomy; however, it is advised that patients undergo D2 lymphadenectomy in a high‐volume surgical center.
  ○ It is recommended that tumors with an expansive growth pattern, including intestinal histotypes, have a proximal margin of ≥3 cm. For tumors with an infiltrative growth pattern, such as poorly cohesive/diffuse histotypes, a proximal margin of ≥5 cm is recommended. When these criteria cannot be met, it is advisable to conduct a thorough examination of the entire thickness of the proximal resection margin using frozen section analysis.

#### 7.2.3 What is the adequate resection margin for patients undergoing gastrectomy?

The sufficient resection margin in patients undergoing gastrectomy depends on the stage and type of the tumor. Different guidelines provide specific recommendations:

- KGCA
  ○ In cases of advanced or infiltrative GC, it is recommended to aim for various macroscopic margin lengths ranging from 3 to 8 cm to ensure pathologically negative resection margins.
  ○ Utilizing intraoperative frozen sections is advised, as it has demonstrated enhanced accuracy in achieving an R0 resection in advanced cancer cases compared with macroscopic margin estimation.
  ○ In advanced stages of the disease (≥ pT3 or ≥pN2 or ≥stage IIIa), obtaining a negative margin is crucial. However, a positive microscopic margin may not have a substantial impact on prognosis.
  ○ In situations where R1 resection occurs in advanced cases, the consideration of reoperation should be approached with caution. Factors such as pathologic stage, patient condition, risk of complications, and potential for delayed systemic therapy should be taken into account.
- JGCA
  ○ For tumors with a depth of T2 or beyond and an expansive growth pattern (types 1 and 2), it is recommended to have a proximal margin of at least 3 cm. In cases where tumors have a depth of T2 or beyond but exhibit an infiltrative growth pattern (types 3 and 4), a proximal margin of 5 cm is advised.
  ○ In situations where achieving these recommended margins proves challenging, it is advisable to thoroughly examine the entire thickness of the proximal resection margin using frozen section analysis.
  ○ For T1 tumors, obtaining a gross resection margin of 2 cm is recommended. In instances where the tumor border is unclear, endoscopic marking of the tumor border with clips based on biopsy results may be beneficial.

#### 7.2.4 What is the recommended extent of the resection of the esophagus and stomach in patients with GEJ cancer?

Different guidelines provide various options:

- KGCA

PG with double tract reconstruction or total gastrectomy are viable options to consider for early GC located in the upper third of the stomach. In cases of advanced GC with adenocarcinoma histology situated in the gastroesophageal junction (Siewert II/III) without serosal invasion, PG may be performed due to the lower incidence of lymph node metastasis in the distal part of the stomach.

- JGCA

The selection of the surgical procedure for GEJ cancer can involve PG with or without lower esophageal resection, total gastrectomy with or without lower esophageal resection, or esophageal resection, and PG. The choice of procedure is contingent on a range of factors and is tailored to the specific patient's health status and tumor attributes.

- NICE
  ○ For individuals diagnosed with localized gastroesophageal junctional adenocarcinoma (excluding T1N0 tumors) who are eligible for surgical resection, two treatment paths are presented:
    - Chemotherapy, either before or both before and after surgery.
    - Chemoradiotherapy, administered prior to surgery.
  ○ The decision between these options is reached after a thorough discussion of the advantages, risks, and potential outcomes of each approach with the individual and their support system. Patients are encouraged to explore participation in pertinent clinical trials if such opportunities are accessible.

#### 7.2.5 What is the role of laparoscopic surgery in GC?

The significance of laparoscopic surgery in GC is firmly established and has been recommended in specific scenarios by various guidelines:

- KGCA
  ○ Laparoscopic distal gastrectomy is advised for cStage I GC due to its superior short‐term surgical results and comparable long‐term survival rates when compared with open distal gastrectomy.
- JGCA
  ○ Laparoscopic distal gastrectomy for cStage I GC is highly recommended as one of the established standard treatments.
  ○ Laparoscopic total gastrectomy or PG, on the other hand, receive a weaker recommendation, suggesting that there is less robust evidence supporting these procedures.
  ○ For cStage II/III GC, specific and clear‐cut recommendations cannot be provided, signifying that the available evidence for laparoscopic surgery in more advanced stages is limited.
- NCCN
  ○ Minimally invasive surgical (MIS) methods, such as laparoscopic or robotic approaches, are available options.
  ○ Both early and locally advanced GCs can be candidates for laparoscopic or robotic gastrectomy, as research from both Eastern and Western studies indicates comparable oncologic outcomes.
  ○ It is generally not advised to employ minimally invasive approaches for T4b (locally advanced) or N2 bulky GC.
- ESMO
  ○ A laparoscopic approach may be recommended selectively in the hands of experienced surgeons.

#### 7.2.6 What is the role of robot‐assisted surgery in GC?

The role of robot‐assisted surgery in GC has been explored in various guidelines, and here are the key points:

- KGCA
  ○ Robotic gastrectomy can be contemplated as a viable treatment option for GC.
- JGCA
  ○ Robot‐assisted surgery receives a weaker recommendation for cStage I GC.
- ESMO
  ○ Robot‐assisted gastrectomy has demonstrated comparable oncological outcomes in terms of survival and lymph node yield compared with conventional laparoscopic gastrectomy.

#### 7.2.7 What is the role of omentectomy in patients with GC?

According to KGCA, in patients with advanced GC, partial omentectomy demonstrates similar survival rates, recurrence rates, and complication rates when compared with total omentectomy. On the other hand, JGCA emphasizes that the removal of the greater omentum is typically part of the standard gastrectomy procedure for T3 or deeper tumors. In cases of T1/T2 tumors, the omentum that is more than 3 cm away from the gastroepiploic artery may be preserved.

#### 7.2.8 What is the role of surgery for metastatic GC?

All guidelines agree that surgery is not the primary approach for metastatic GC due to the advanced stage of the disease. KGCA discourages upfront gastrectomy and emphasizes the role of chemotherapy. ESMO suggests gastrectomy only for palliation of symptoms. KGCA suggests conversion surgery as an option for patients with limited metastasis and a good chemotherapy response. This aligns with JGCA and ESMO's stance on considering surgery in specific cases with favorable responses to chemotherapy. KGCA and JGCA both mention the consideration of radical gastrectomy and metastasectomy for selected patients with liver or ovarian oligometastases, respectively. ESMO also mentions a potential role for resection in cases of oligometastatic disease and positive chemotherapy response.

### 7.3 Surgery (lymphadenectomy)

#### 7.3.1 What is the definition of the extent of systematic lymphadenectomy in patients with GC?

Based on the information from different guidelines the extent of systematic lymphadenectomy is:

- JGCA
  ○ The scope of systematic lymphadenectomy is determined by the type of gastrectomy performed, including total gastrectomy, distal gastrectomy, PPG, and PG.
  ○ In the case of total gastrectomy, D1 involves the removal of lymph nodes in stations 1–7, D1+ encompasses D1 nodes plus nodes 8a, 9, and 11p. D2 extends further by including nodes 8a, 9, 11p, 11d, and 12a in addition to D1.
  ○ In situations where tumors invade the esophagus, resection of nodes 19, 20, and 110 should be included in the D2 lymphadenectomy.
  ○ For distal gastrectomy, D1 entails the removal of lymph nodes in stations 1, 3, 4sb, 4d, 5, 6, and 7. D1+ encompasses D1 nodes plus nodes 8a and 9. D2 further includes nodes 8a, 9, 11p, and 12a in addition to D1.
- NCCN
  ○ D1 dissection entails the removal of lymph nodes along the right and left cardiac, lesser and greater curvature, suprapyloric along the right gastric artery, and infrapyloric area.
  ○ D2 dissection encompasses all the nodes from D1, in addition to those along the left gastric artery, common hepatic artery, celiac artery, and splenic artery.

#### 7.3.2 What criteria dictate the need for lymph node dissection in patients with GC, considering their clinical stage?

Based on the guidelines, the indications to perform lymph node dissection based on clinical stage in patients with GC are as follows:

- JGCA
  ○ D2 lymphadenectomy is recommended for cN+ or ≥cT2 tumors.
  ○ cT1N0 tumors are indicated for either D1 or D1+ lymphadenectomy.
  ○ D1 lymphadenectomy is suggested for cT1a tumors not meeting EMR or ESD criteria and for cT1bN0 tumors of differentiated type and 1.5 cm or smaller in diameter.
  ○ D1+ lymphadenectomy is advised for cT1N0 tumors that do not meet the criteria for D1 lymphadenectomy.
  ○ Potentially curable cT2–T4 tumors and cT1N+ tumors should undergo D2 lymphadenectomy.
  ○ In total gastrectomy for advanced cancer of the proximal stomach, spleen preservation is recommended provided the tumor does not involve the greater curvature.
- NCCN
  ○ During gastric resection, it is recommended to perform either D1 or D2 lymphadenectomy, which entails the removal of perigastric lymph nodes (D1) and those located along the named vessels of the celiac axis (D2).
  ○ The objective is to thoroughly examine a minimum of 16 or more lymph nodes during the lymphadenectomy procedure.
- NICE
  ○ When conducting a curative gastrectomy for individuals with GC, it is advisable to contemplate a D2 lymph node dissection.

#### 7.3.3 What are the indications for extended lymphadenectomy beyond D2 in GC?

Based on the guidelines, the indications for extended lymphadenectomy beyond D2 in GC are as follows:

- KGCA
  ○ Prophylactic splenectomy for dissection of splenic hilar lymph nodes (No. 10) is discouraged in cases of curative resection for advanced GC in the proximal stomach without invasion of the greater curvature.
  ○ Therapeutic splenectomy might be warranted if the tumor directly infiltrates the spleen or if there is suspicion of lymph node metastasis around the splenic hilum.
- JGCA
  ○ Indications for extended lymphadenectomy beyond include:
  ○ For cancer of the proximal stomach invading the greater curvature (D2 + No. 10), dissection of No. 10 (splenic hilar lymph nodes) may be performed, with or without splenectomy.
  ○ In cases of cancer of the distal stomach with metastasis to the No. 6 lymph nodes (D2 + No. 14v), dissection of No. 14v (superior mesenteric venous lymph node) is indicated.
  ○ For cancer invading the duodenum (D2 + No. 13), dissection of No. 13 (posterior pancreas head lymph node) is recommended.
  ○ After neoadjuvant chemotherapy for cancer with extensive lymph node involvement (D2 + No. 16), dissection of No. 16 (abdominal aortic lymph node) should be considered.
- NCCN
  ○ Splenectomy is typically not performed routinely, unless there is direct involvement of the spleen or extensive adenopathy is observed in the hilar region

#### 7.3.4 What is the advised scope of lymphadenectomy for individuals with GEJ

Based on the guidelines, the recommended extent of lymphadenectomy in patients with GEJ is as follows:

- KGCA
  ○ In cases of early GEJ cancers, lower mediastinal lymph node dissection may not be deemed necessary.
  ○ However, for advanced GEJ cancer with esophageal involvement exceeding 2 cm, it may be crucial to include lower mediastinal lymph node dissection in order to thoroughly remove potential metastatic lymph nodes.
  ○ The transhiatal approach is advised to achieve a negative resection margin and perform lower mediastinal lymph node dissection in resectable adenocarcinoma invading the GEJ.
- JGCA
  ○ For cT2‐T4 junctional cancer with clinically positive nodes in the upper/middle mediastinal field, lymph node dissection of stations 1, 2, 3a, 7, 8a, 9, 11p, 106recR, 107, 108, 109, 110, 111, and 112 through the right transthoracic approach is recommended.
  ○ For cT2‐T4 junctional cancer clinically node‐negative based on esophageal infiltration length, the recommended lymph node dissection is as follows:
  ○ For ≤2.0 cm esophageal infiltration, dissection of stations 1, 2, 3a, 7, 8a, 9, 11p, and 19.
  ○ For 2.1–4.0 cm esophageal infiltration, dissection of stations 1, 2, 3a, 7, 8a, 9, 11p, 19, and 110.
  ○ For >4 cm esophageal infiltration, dissection of stations 1, 2, 3a, 7, 8a, 9, 11p, 19, 106recR, 107, 108, 109, 110, 111, and 112 through the transhiatal approach is recommended.

### 7.4 Systemic chemotherapy

#### 7.4.1 What are the established patient criteria for undergoing systemic chemotherapy in GC?

The criteria for initiating systemic chemotherapy in GC are uniform across the guidelines. They suggest systemic chemotherapy for individuals with advanced GC or those who have undergone R2 resection, provided their overall health and major organ functions are maintained. The decision to offer chemotherapy should take into account the patient's performance status, medical conditions, and ability to tolerate specific treatment regimens. Furthermore, the use of targeted therapies, such as trastuzumab for HER2‐positive adenocarcinoma, is advised in suitable cases. Perioperative chemotherapy is strongly recommended for patients with resectable GC, whereas the consideration of postoperative chemotherapy or chemoradiation depends on factors like the extent of lymph node dissection and other clinical aspects.

#### 7.4.2 What is the recommended adjuvant chemotherapy regimen in GC?

The recommended adjuvant chemotherapy regimens for GC based on different guidelines are as follows:

- KGCA
  ○ Patients diagnosed with pathological stage II or III GC are advised to undergo adjuvant chemotherapy, with either S‐1 or XELOX regimens being the recommended options.
- JGCA
  ○ Patients with pathological stage II or III GC (pStage II or III) are advised to undergo adjuvant chemotherapy, which may consist of S‐1 alone or in combination therapy.
  ○ Postoperative chemotherapy for patients with curatively resected stage IV GC is suggested, although with a lower level of recommendation.
- NCCN
  ○ Patients who have undergone primary D2 lymph node dissection are recommended to undergo postoperative chemotherapy.
  ○ The preferred regimens for postoperative chemotherapy include either capecitabine and oxaliplatin or fluorouracil and oxaliplatin.
  ○ Postoperative chemoradiation is recommended for patients who received less than a D2 lymph node dissection.
  ○ Fluoropyrimidine‐based treatment (either infusional fluorouracil or capecitabine) is administered both before and after fluoropyrimidine‐based chemoradiation.
- ESMO
  ○ Adjuvant chemotherapy is advised, consisting of a doublet regimen lasting for a total of 6 months. This regimen should include a fluoropyrimidine (such as fluorouracil or capecitabine) along with either oxaliplatin or docetaxel.
  ○ Patients with stage ≥IB GC who have undergone surgery without prior administration of preoperative chemotherapy are recommended to receive adjuvant chemotherapy.
  ○ For patients who have not received preoperative chemotherapy and have not undergone an appropriate D2 lymphadenectomy, adjuvant chemoradiotherapy can be considered as a potential option.
  ○ In cases where patients have undergone surgery with involved margins (R1), adjuvant radiation therapy or chemoradiotherapy may be considered on an individual basis, but it is not considered standard practice.
  ○ For patients with MSI‐H GC who have undergone curative surgery, adjuvant chemotherapy is not a recommended course of action. However, if a response is needed to downstage a tumor before surgery, the fluorouracil, leucovorin, oxaliplatin, and docetaxel (FLOT) regimen is advised.

#### 7.4.3 What are the recommended first line chemotherapy, targeted therapy and immunotherapy in unresectable locally advanced, recurrent or metastatic GC?

The suggested first‐line chemotherapy, targeted therapy, and immunotherapy for unresectable locally advanced, recurrent, or metastatic GC are as follows based on different guidelines:

- KGCA
  ○ For individuals with locally advanced unresectable or metastatic GC, the initial palliative treatment of choice is platinum/fluoropyrimidine‐based chemotherapy.
  ○ Patients with advanced GC who exhibit HER2 IHC 3+ or IHC 2+ with ISH‐positive status are advised to undergo palliative first‐line treatment involving trastuzumab in combination with capecitabine or fluorouracil (FU) along with cisplatin.
  ○ Palliative first‐line therapy comprising nivolumab in combination with capecitabine or FU plus oxaliplatin (XELOX or FOLFOX) is recommended for patients with PD‐L1 CPS ≥5 and HER2‐negative advanced GC.
- JGCA
  ○ In cases of HER2‐negative GC, various chemotherapy regimens are suggested. These may include combinations such as S‐1 and oxaliplatin, capecitabine and oxaliplatin, 5‐FU/levofolinate calcium with oxaliplatin, and tegafur/5‐chloro 2, 4‐dihydroxypyridine/potassium oxonate (S‐1) in conjunction with cisplatin, among others.
  ○ For HER2‐positive GC, recommended treatment regimens encompass combinations of capecitabine or S‐1 and cisplatin along with trastuzumab.
- NCCN
  ○ When considering first‐line therapy, oxaliplatin is generally favored over cisplatin due to its lower associated toxicity.
  ○ For cases of HER2‐positive adenocarcinoma, the preferred treatment regimens comprise a combination of fluoropyrimidine (either fluorouracil or capecitabine), oxaliplatin, and trastuzumab, or a combination of fluoropyrimidine (either fluorouracil or capecitabine), cisplatin, and trastuzumab.
  ○ In cases where HER2 is not overexpressed, recommended regimens include a combination of fluoropyrimidine (either fluorouracil or capecitabine), oxaliplatin, and nivolumab (for cases with PD‐L1 CPS ≥5), or a combination of fluoropyrimidine (either fluorouracil or capecitabine) and oxaliplatin or cisplatin.
- ESMO
  ○ The initial line of treatment typically involves a combination of platinum and fluoropyrimidine, with a preference for oxaliplatin, particularly in older patients. S‐1 is a commonly administered option for Asian patients.
  ○ For individuals with HER2‐positive tumors, treatment involving trastuzumab in conjunction with chemotherapy is recommended.
  ○ In cases of advanced, untreated gastric, OGJ, and esophageal cancer with a PD‐L1 CPS ≥5, nivolumab‐based chemotherapy is advised.
- NICE
  ○ Trastuzumab, when combined with cisplatin and either capecitabine or 5‐fluorouracil, is recommended as a viable treatment option for individuals diagnosed with HER2‐positive metastatic adenocarcinoma of the stomach or gastro‐esophageal junction.
  ○ For those with advanced oesophago‐GC and a performance status of 0 to 2, as well as no significant comorbidities, the preferred approach is first‐line palliative combination chemotherapy. Possible drug combinations encompass doublet treatment (involving either 5‐fluorouracil or capecitabine combined with cisplatin or oxaliplatin) or triplet treatment (involving either 5‐fluorouracil or capecitabine combined with cisplatin or oxaliplatin plus epirubicin).

#### 7.4.4 What are the recommended second and later line chemotherapy, targeted therapy and immunotherapy in unresectable locally advanced, recurrent or metastatic GC?

The recommended second and later‐line chemotherapy, targeted therapy, and immunotherapy for unresectable locally advanced, recurrent, or metastatic GC are as follows based on different guidelines:

- KGCA
  ○ While the preferential recommendation is for the combination of ramucirumab and paclitaxel, other agents may also be considered as potential treatment options.
- JGCA
  ○ Patients with an adequate performance status are advised to undergo second‐line treatment. For patients with microsatellite instability‐high (MSI‐H) GC, pembrolizumab and paclitaxel plus ramucirumab is recommended. For non‐MSI‐High patients, paclitaxel plus ramucirumab is recommended.
  ○ Patients in good overall condition following the completion of second‐line treatment should contemplate third‐line treatment. Monotherapy with nivolumab, irinotecan, or trifluridine/tipiracil is the recommended regimen for third‐ or later‐line treatment for patients with good general condition.
- NCCN
  ○ The choice of second‐line or subsequent therapy is contingent on prior treatment and the patient's performance status.
  ○ Preferred regimens encompass ramucirumab and paclitaxel, Fam‐trastuzumab deruxtecan‐nxki for HER2 overexpression positive adenocarcinoma, Docetaxel, Paclitaxel, Irinotecan, Fluorouracil and irinotecan, and Trifluridine and tipiracil. Additionally, other recommended regimens include Ramucirumab, Irinotecan and cisplatin, Fluorouracil and irinotecan with ramucirumab, Irinotecan and ramucirumab, and Docetaxel and irinotecan.
  ○ Entrectinib or larotrectinib demonstrate efficacy in NTRK gene fusion‐positive tumors.
  ○ Pembrolizumab is an effective option for MSI‐H or dMMR tumors, as well as for TMB high (≥10 mutations/megabase) tumors. Dostarlimab‐gxly is beneficial for MSI‐H or dMMR tumors.
- ESMO
  ○ Ramucirumab‐paclitaxel is a favored choice for second‐line treatment in GC. Ramucirumab as a standalone therapy is also a viable option.
  ○ Pembrolizumab is the recommended second‐line treatment for patients with MSI‐H/dMMR GC.
  ○ Trastuzumab deruxtecan may be considered for HER2‐positive advanced GC after first‐line therapy.
- NICE
  ○ When it comes to second‐line palliative chemotherapy for oesophago‐GC, open communication with the patient and their caregivers is essential, weighing the potential risks, benefits, treatment outcomes, and taking patient preferences into account.
  ○ For eligible patients, clinical trials could be a viable option as an alternative to second‐line chemotherapy.

#### 7.4.5 What is the role neoadjuvant chemotherapy in patients with GC?

The role of neoadjuvant chemotherapy in patients with GC is as follows:

- KGCA
  ○ Neoadjuvant chemotherapy, integrated into perioperative treatment, may be contemplated for patients with surgically manageable locally advanced GC.
- JGCA
  ○ The efficacy of neoadjuvant chemotherapy has been consistently demonstrated in studies from Western nations, China, and Korea. Nevertheless, in Japan, while positive outcomes have been observed in specific instances, there is no unanimous agreement regarding its extensive application.
- NCCN
  ○ Perioperative chemotherapy is preferably administered through the FLOT regimen or fluoropyrimidine and oxaliplatin.
  ○ Another recommended option is fluorouracil and cisplatin.
  ○ In terms of chemoradiation, while there is not a specific preference, suggested regimens encompass paclitaxel and carboplatin, fluorouracilb and oxaliplatin, fluorouracil and cisplatin, or fluoropyrimidine (fluorouracil or capecitabine). Infusional fluorouracilb can be substituted with capecitabine.

### 7.5 Radiotherapy

#### 7.5.1 What is the role of radiation therapy in patients with GC?

The role of radiation therapy in patients with GC is as follows:

- KGCA
  ○ Including adjuvant chemo‐radiation therapy is typically not advised for patients with pathological stage II or III GC who have undergone complete resection with D2 lymphadenectomy.
  ○ The available evidence regarding the addition of radiation to neoadjuvant chemo‐radiation therapy in patients with locally advanced GC is inconclusive.
- NCCN
  ○ Siewert I and II tumors should be treated following radiation therapy guidelines that are relevant to esophageal and GEJ cancers. Depending on the clinical scenario, Siewert III tumors might be better addressed by radiation therapy guidelines applicable to either esophageal and EGJ or GCs, and this decision may be adjusted based on the location of the primary tumor mass.

### 7.6 Hyperthermic intraperitoneal chemotherapy

#### 7.6.1 What is the role of HIPEC in patients with GC?

Here are the considerations from different guidelines:

- KGCA

For patients diagnosed with GC and peritoneal carcinomatosis, supplementary intraperitoneal chemotherapy may be considered for investigational purposes.

- NCCN

Positive peritoneal cytology, even in the absence of visible peritoneal implants, is indicative of poor prognosis and is classified as pM1 disease. Hyperthermic intraperitoneal chemotherapy (HIPEC) or laparoscopic HIPEC may be contemplated as a therapeutic option for meticulously chosen stage IV patients, within the framework of ongoing clinical trials. The efficacy and safety of HIPEC in patients with GC and peritoneal metastasis are presently being subjected to further clinical scrutiny.

- ESMO

Individuals with confined peritoneal metastases could be evaluated as suitable candidates for cytoreductive surgery along with HIPEC. Nevertheless, it is crucial to emphasize that HIPEC for GC has not yet been firmly established as a standard treatment protocol. Patients with positive lavage cytology are considered uncertain candidates for surgically intended curative resection and are best managed within the framework of a clinical trial.

## 8 SUPPORTIVE AND PALLIATIVE CARE

### 8.1 What is the recommended supportive and palliative care in GC?

Here are the key aspects of supportive and palliative care based on the guidelines:

- NCCN

For patients with incurable GC, gastric resections should be limited to addressing symptomatic issues like obstruction or unmanageable bleeding. Lymph node dissection is not necessary in this palliative context. In cases where surgery is appropriate and the patient has a reasonable prognosis and gastric outlet obstruction, gastrojejunostomy (whether open or laparoscopic) is the preferred approach over endoluminal stenting. As part of palliative care, the placement of a venting gastrostomy and/or feeding tube may also be considered.

- ESMO

Stenting is indicated for patients experiencing severe dysphagia, particularly those with a limited life expectancy. This is because stenting provides an immediate improvement in swallowing, whereas the relief of symptoms from radiotherapy takes time.

- NICE

In cases of esophageal and gastro‐esophageal junctional cancer, self‐expanding stents are a viable option for individuals in need of immediate relief from dysphagia. The choice between stenting and radiotherapy as primary treatment should be guided by the severity of dysphagia and its impact on nutritional status, quality of life, performance status, and prognosis. Moreover, specialized dietary guidance from a dedicated cancer dietitian is recommended for those with oesophago‐GC under palliative care. This dietary support should be individualized based on the patient's specific clinical circumstances and should be coordinated with the multidisciplinary team as well as both hospital and community‐based palliative care teams.

## 9 FOLLOW UP

### 9.1 What is the recommended follow up program for patients with GC?

The recommended follow‐up program for patients with GC may vary slightly depending on the guidelines and the stage of the disease:

- KGCA
  ○ For patients with stage I tumors, a follow‐up plan includes: Physical examination and blood tests, including tumor markers, every 6 months for the first 3 years, and then every 6–12 months up to 5 years postoperatively.
  ○ Abdomen pelvis CT and chest x‐ray every 6 months for 2 years, followed by checks every 6 or 12 months in the third year, and then annually until 5 years postoperatively.
  ○ Esophagogastroduodenoscopy (EGD) once or twice within the first year, and then annually up to 5 years postoperatively, irrespective of the stage.
  ○ After 5 years, all patients should undergo annual EGD, and a routine chest CT scan can be included in the annual follow‐up examinations.
- JGCA
  ○ All patients should have follow‐up for a minimum of 5 years post‐surgery.
  ○ For stage I patients, regular check‐ups including medical examination, performance assessment, body weight monitoring, and blood tests (including tumor markers) are advised every 6 months for the first 2.5 years, followed by annual check‐ups. Abdominal CT and/or ultrasonography should be performed annually. Endoscopy is recommended at 1, 3, and 5 years after initial treatment.
  ○ For stage II and III patients, more frequent monitoring is recommended. This includes check‐ups every 3 months for the first 2 years, followed by check‐ups every 6 months for the subsequent 3 years. Abdominal CT and/or ultrasonography should be conducted every 6 months for the first 3 years, and then annually for the next 2 years. Endoscopy is advised at 1, 3, and 5 years post initial treatment.
- NCCN
  ○ Medical examination, performance status, body weight, blood tests, and imaging (CT chest/abdomen/pelvis) are recommended every few months in the first years after surgery, and then every 6–12 months for the following years.
  ○ Endoscopy is also recommended at specific intervals based on the stage of the disease and type of treatment.
- ESMO
  ○ Regular follow‐up is crucial for detecting signs of disease progression in advanced cases. Radiological examinations, particularly CT scans of the chest and abdomen, should be conducted every 6–12 weeks for patients eligible for additional cancer‐specific treatments. This ensures timely intervention before significant clinical deterioration occurs.
- NICE
  ○ Routine clinical follow‐up and radiological surveillance for the sole purpose of detecting recurrent disease are not recommended for individuals who exhibit no symptoms or evidence of residual disease after curative treatment. Instead, it is advised to educate patients about the potential symptoms of recurrent disease and ensure they have swift access to the multidisciplinary team for review in case such symptoms arise. This approach is more effective in managing post‐treatment care.

## 10 DISCUSSION

The management of GC involves a comprehensive approach aimed at diagnosing, treating, and providing supportive care to patients. In this context, two sets of guidelines stand out: the Eastern guidelines represented by the KGCA and JGCA, and the Western guidelines represented by the NCCN, ESMO, BSG, and NICE. If we look at the approach each set has generally we may summarize the difference as follow based on different aspects of management of GC:

(1) Genetic predisposition: Both Eastern and Western guidelines acknowledge the role of genetic predisposition, particularly in hereditary diffuse GC (HDGC) associated with CDH1 gene mutations. Although the Eastern guidelines emphasize genetic counseling and testing for patients with family history, the Western guidelines provide comprehensive recommendations on genetic testing, risk assessment, and management for individuals with suspected or confirmed CDH1 mutations, advocating for prophylactic gastrectomy.
(2) Prevention: The Eastern guidelines underscore the significance of *H. pylori* eradication as a preventive measure for GC, particularly in high‐risk populations. They advocate for testing and treating *H. pylori* infection, especially in patients with atrophic gastritis or intestinal metaplasia. In contrast, Western guidelines recommend *H. pylori* testing and treatment primarily in cases of specific indications such as peptic ulcer disease, without placing the same emphasis on its role in preventing GC. This difference in approach reflects differing levels of *H. pylori* prevalence and disease burden in various regions.
(3) Management of gastric polyps, atrophy, dysplasia, and metaplasia: Both sets of guidelines recognize the significance of gastric polyps, atrophy, dysplasia, and metaplasia as precursor lesions for GC. However, the Eastern guidelines tend to provide more specific recommendations for surveillance intervals and management strategies based on the degree of dysplasia. The Western guidelines also acknowledge the importance of surveillance and management but may offer a broader range of options, including endoscopic resection for certain polyps and close monitoring for lesions with LGD.
(4) Diagnosis: Both Eastern and Western guidelines advocate for endoscopic assessment for diagnosing GC and its precursor lesions. However, the Eastern guidelines, particularly JGCA, place greater emphasis on magnifying endoscopy with narrow‐band imaging (M‐NBI) for early detection of lesions, as it has demonstrated high sensitivity in identifying superficial neoplastic changes. The Western guidelines stress the importance of endoscopic techniques such as chromoendoscopy and high‐definition imaging for accurate diagnosis.
(5) Pathology and molecular biology: Both perspectives recognize the significance of Lauren classification and molecular subtypes in understanding GC pathology. However, the Eastern guidelines delve into more specific molecular markers and provide guidance on their clinical relevance, such as HER2 status for targeted therapy. The Western guidelines similarly emphasize molecular testing, including HER2 and PD‐L1, for treatment decisions but may include additional markers like MSI‐H and EBV.
(6) Treatment: In the realm of surgery, the guidelines from both Eastern and Western perspectives recommend radical gastrectomy with extended lymphadenectomy for resectable GC. However, the Eastern guidelines, represented by KGCA and JGCA, tend to emphasize D2 lymph node dissection, a more aggressive approach involving the removal of a higher number of lymph nodes. This approach is supported by studies showing potential survival benefits, particularly in Asian populations.
  On the other hand, the Western guidelines, such as NCCN, ESMO, BSG, and NICE, acknowledge D2 lymphadenectomy as an option but lean more toward D1 lymph node dissection, which removes fewer lymph nodes and may have a reduced risk of surgical complications. This difference highlights the varying interpretations of the balance between potential survival benefits and surgical morbidity, reflecting diverse clinical practices, and regional preferences.
  The recommended adjuvant chemotherapy regimens also exhibit differences between the Eastern and Western guidelines. The Eastern guidelines, specifically KGCA and JGCA, recommend S‐1 as a preferred adjuvant chemotherapy option for patients with pathological stage II or III GC. This regimen has shown efficacy in Asian populations, showcasing regional variations in treatment options. Conversely, the Western guidelines, including NCCN, ESMO, BSG, and NICE, advocate for a broader range of regimens involving fluoropyrimidine‐based combinations with oxaliplatin or other agents. There is less consensus on a single preferred regimen, reflecting the consideration of patient characteristics and individualized treatment decisions. This divergence underscores the influence of regional factors, including drug availability, patient populations, and clinical trial data.
  The approach to neoadjuvant chemotherapy further highlights the distinction between Eastern and Western guidelines. While both perspectives recognize its potential benefits in improving the curative resection rate, the Eastern guidelines, particularly from JGCA, express reservations regarding the widespread use of neoadjuvant chemotherapy. This hesitation may be attributed to variations in trial data, patient demographics, and surgical practices across different regions. In contrast, the Western guidelines, such as NCCN, ESMO, BSG, and NICE, endorse neoadjuvant chemotherapy with specific regimens such as FLOT or fluoropyrimidine‐based combinations. This discrepancy mirrors the differing levels of confidence and adoption in neoadjuvant approaches, potentially driven by variations in disease presentation and patient characteristics.
(7) Supportive and palliative care: Both sets of guidelines emphasize the importance of supportive and palliative care for improving patients' quality of life. The Eastern guidelines, especially KGCA, underscore tailored nutritional support, pain management, and psychological support. The Western guidelines offer comprehensive recommendations for symptom management, nutritional support, and psychological interventions, highlighting the multidisciplinary nature of care and the importance of addressing both physical and emotional needs.
(8) Follow‐up: Both Eastern and Western guidelines emphasize follow‐up programs to monitor for recurrence and provide appropriate care. However, the Eastern guidelines provide more specific intervals for follow‐up visits, imaging, and endoscopy. The Western guidelines stress individualized follow‐up plans and highlight the importance of rapid access to multidisciplinary teams for symptom management and addressing complications.

**TABLE 2:** Summary of similarities and differences between Eastern and Western guidelines.

Aspect	Similarities	Differences
Genetic predisposition	Acknowledge the role of genetic predisposition, particularly in hereditary diffuse GC associated with CDH1 gene mutations.	Eastern guidelines emphasize genetic counseling and testing, whereas Western guidelines provide comprehensive recommendations on genetic testing, risk assessment, and advocate for prophylactic gastrectomy.
Prevention	Recognize the significance of *H. pylori* infection in GC.	Eastern guidelines emphasize *H. pylori* eradication, especially in high‐risk populations, whereas Western guidelines recommend testing and treatment primarily in specific indications like peptic ulcer disease. Less emphasis on prevention.
Management of precursor lesions	Acknowledge the significance of gastric polyps, atrophy, dysplasia, and metaplasia as precursor lesions for GC.	Eastern guidelines provide specific recommendations for surveillance intervals based on the degree of dysplasia, whereas Western guidelines acknowledge the importance but offer a broader range of options, including endoscopic resection.
Diagnosis	Advocate endoscopic assessment for diagnosing GC and its precursor lesions.	Eastern guidelines, particularly JGCA, place greater emphasis on magnifying endoscopy with NBI for early detection, whereas Western guidelines stress the importance of endoscopic techniques such as chromoendoscopy and high‐definition imaging.
Pathology and molecular biology	Recognize the significance of Lauren classification and molecular subtypes in understanding GC pathology.	Eastern guidelines delve into more specific molecular markers and provide guidance on their clinical relevance. The Western guidelines include additional markers like MSI‐H and EBV.
Surgery and lymphadenectomy	Recommend radical gastrectomy with extended lymphadenectomy for resectable GC.	Eastern guidelines tend to emphasize D2 lymph node dissection, a more aggressive approach involving the removal of a higher number of lymph nodes. Western guidelines acknowledge D2 but lean more toward D1 lymph node dissection, which removes fewer lymph nodes and may have a reduced risk of surgical complications.
Adjuvant chemotherapy	Emphasize the importance of adjuvant chemotherapy for specific stages of GC.	Eastern guidelines recommend S‐1 as a preferred adjuvant chemotherapy option for patients with pathological stage II or III GC, reflecting regional variations. Western guidelines advocate for a broader range of regimens involving fluoropyrimidine‐based combinations with oxaliplatin or other agents, showing less consensus on a single preferred regimen.
Neoadjuvant chemotherapy	Recognize the potential benefits of neoadjuvant chemotherapy in improving the curative resection rate.	Eastern guidelines express reservations regarding the widespread use, potentially attributed to variations in trial data, patient demographics, and surgical practices across different regions. Western guidelines endorse neoadjuvant chemotherapy with specific regimens (e.g., FLOT) and show confidence in its adoption, potentially driven by variations in disease presentation and patient characteristics.
Supportive and palliative care	Emphasize the importance of supportive and palliative care for improving patients' quality of life.	The difference lies in the level of specificity, with Eastern guidelines emphasizing a tailored approach and Western guidelines providing a more comprehensive set of recommendations for supportive and palliative care in GC management.
Follow‐up	Stress the importance of follow‐up programs to monitor for recurrence and provide appropriate care.	Eastern guidelines provide more specific intervals for follow‐up visits, imaging, and endoscopy. Western guidelines stress individualized follow‐up plans and highlight the importance of rapid access to multidisciplinary teams for symptom management and addressing complications.

*Note*: Eastern guidelines include KGCA and JGCA and Western guidelines include NCCN, ESMO, BSG, and NICE.

Abbreviations: EBV, Epstein–Barr virus; FLOT, fluorouracil, leucovorin, oxaliplatin, and docetaxel; GC, gastric cancer; MSI‐H, microsatellite instability‐high; NBI, narrow‐band imaging.

Summary of discussed subjects can be found in the following table Table 2.

Pan‐Asian^18^ experts undertook the task of adapting the ESMO Clinical Practice Guidelines, last updated in July 2023, to reflect the consensus opinions of a panel representing oncological societies from various Asian countries. These societies include China (CSCO), Indonesia (ISHMO), India (ISMPO), Japan (JSMO), Korea (KSMO), Malaysia (MOS), the Philippines (PSMO), Singapore (SSO), Taiwan (TOS), and Thailand (TSCO). The coordination of this effort was carried out by ESMO and the Japanese Society of Medical Oncology (JSMO). In the preliminary survey before the meeting, 20 voting Asian experts assessed the “acceptability” of 38 recommendations from the latest ESMO Clinical Practice Guidelines concerning the diagnosis, treatment, and follow‐up of GC patients. Discrepancies in agreement emerged for 23 recommendations, with 16 of them discussed during a face‐to‐face working meeting in Tokyo. The discrepancies primarily pertained to the “applicability” of the recommendations in specific Asian countries/regions rather than their “scientific acceptability.” The results of voting, both before and after the Tokyo meeting, demonstrated over 80% concordance with ESMO recommendations for GC treatment. Following the face‐to‐face discussions, adjustments were made to the topic of debates including use of diagnostic laparoscopy, indications and regimens of preoperative chemotherapy, adjuvant chemotherapy for MSI‐H patients, first‐line chemotherapy in cases of platinum compounds intolerance and indication of adding zolbetuximab, first‐line chemotherapy in PD‐L1p‐positive cases, indication for trastuzumab deruxtecan usage and the use of low‐dose olanzapine as dietary support. Also, two recommendations were deleted from original ESMO; “use of preoperative chemotherapy for IB‐III GC” and “use of embrolizumab for patients with adenocarcinoma of the esophagus and esophago‐gastric junction expressing PD‐L1 CPS ≥1.” Therefore, Pan‐Asian authors achieved a 100% consensus on the “acceptability” of ESMO recommendations. Notably, variations in the availability of diagnostic testing and treatment options among patients in different regions were attributed to differences in healthcare system organization and reimbursement strategies, impacting the implementation of scientific recommendations in those regions.

## 11 CONCLUSION

In conclusion, a comparison of Eastern and Western guidelines for GC management reveals a blend of shared priorities and regional distinctions. Both sets of guidelines prioritize comprehensive diagnostic evaluations, personalized treatment plans, and palliative care. However, variations arise in treatment regimens, the adoption of newer therapies like immunotherapy, and the utilization of emerging techniques such as HIPEC. These differences reflect the diverse clinical landscapes, research focuses, and healthcare systems within these regions. Ultimately, the harmonization of these guidelines can provide a holistic approach to managing GC, catering to the unique needs of patients across different parts of the world.

## AUTHOR CONTRIBUTIONS

**Amirmohsen Jalaeefar:** Conceptualization (lead); investigation (lead); methodology (lead); project administration (lead); supervision (lead); writing – review and editing (lead). **Seyedeh Zahra Mousavi:** Conceptualization (lead); investigation (lead); methodology (lead); project administration (lead); supervision (lead); writing – review and editing (lead). **Mohammad Shirkhoda:** Conceptualization (equal); formal analysis (equal); methodology (equal); software (equal); validation (equal). **Habibollah Mahmoodzadeh:** Data curation (equal); formal analysis (equal); software (equal); validation (equal); visualization (equal); writing – original draft (equal). **Narjes Mohammadzadeh:** Formal analysis (equal); resources (equal); software (equal); supervision (equal); validation (equal); visualization (equal). **Amirsina Sharifi:** Data curation (equal); formal analysis (equal); investigation (equal); project administration (equal); supervision (equal); writing – original draft (equal); writing – review and editing (equal).

## CONFLICT OF INTEREST STATEMENT

The authors have stated explicitly that there are no conflicts of interest in connection with this article.

## ETHICS STATEMENT

Our recent study did not involve human subjects and used only pre‐published guidelines. As such, we did not require ethics committee certification.

## Data Availability

Data sharing is not applicable to this article as no new data were created or analyzed in this study.
